# Supplementary material for: Time-series transcriptome analysis identified differentially expressed genes in broiler chicken infected with mixed Eimeria species
Source: Front Genet. 2022 Aug 8;13:886781. doi: 10.3389/fgene.2022.886781 (PMC9393255; doi:10.3389/fgene.2022.886781)
Supplement: Supplementary file 2 [file DataSheet1.ZIP › 7dpi_GO.Gsea.1625106495923/GOBP_EMBRYONIC_AXIS_SPECIFICATION.html]

Details for gene set GOBP\_EMBRYONIC\_AXIS\_SPECIFICATION[GSEA]

|  || Dataset | TMM\_7dpi\_gct\_format\_7dpi\_gct\_format.Class\_4dpi.cls #PC\_versus\_NC.Class\_4dpi.cls #PC\_versus\_NC\_repos |
| Phenotype | Class\_4dpi.cls#PC\_versus\_NC\_repos |
| Upregulated in class | 0 |
| GeneSet | GOBP\_EMBRYONIC\_AXIS\_SPECIFICATION |
| Enrichment Score (ES) | -0.6998641 |
| Normalized Enrichment Score (NES) | -1.8201134 |
| Nominal p-value | 0.0018177096 |
| FDR q-value | 0.10645763 |
| FWER p-Value | 0.9086 |
Table: GSEA Results Summary

  

Fig 1: Enrichment plot: GOBP\_EMBRYONIC\_AXIS\_SPECIFICATION      
 Profile of the Running ES Score & Positions of GeneSet Members on the Rank Ordered List

  

| SYMBOL | TITLE | RANK IN GENE LIST | RANK METRIC SCORE | RUNNING ES | CORE ENRICHMENT || 1 | WNT1 | na | 1130 | 0.474 | -0.0500 | No |
| 2 | WNT5A | na | 2943 | 0.133 | -0.1886 | No |
| 3 | SMAD6 | na | 3601 | 0.052 | -0.2385 | No |
| 4 | TMED2 | na | 3792 | 0.029 | -0.2516 | No |
| 5 | WDR77 | na | 4212 | -0.019 | -0.2848 | No |
| 6 | HOXD8 | na | 7354 | -0.347 | -0.5142 | No |
| 7 | PLD6 | na | 9272 | -0.594 | -0.6186 | No |
| 8 | EPB41L5 | na | 10248 | -0.757 | -0.6292 | Yes |
| 9 | COBL | na | 10610 | -0.835 | -0.5814 | Yes |
| 10 | STIL | na | 10740 | -0.863 | -0.5116 | Yes |
| 11 | PTCH1 | na | 10981 | -0.931 | -0.4448 | Yes |
| 12 | FRS2 | na | 11717 | -1.256 | -0.3888 | Yes |
| 13 | TBX3 | na | 11786 | -1.314 | -0.2719 | Yes |
| 14 | BASP1 | na | 11851 | -1.421 | -0.1447 | Yes |
| 15 | TBXT | na | 11959 | -1.693 | 0.0043 | Yes |
Table: GSEA details [plain text format]

  

Fig 2: GOBP\_EMBRYONIC\_AXIS\_SPECIFICATION      
 Blue-Pink O' Gram in the Space of the Analyzed GeneSet

  

Fig 3: GOBP\_EMBRYONIC\_AXIS\_SPECIFICATION: Random ES distribution      
 Gene set null distribution of ES for **GOBP\_EMBRYONIC\_AXIS\_SPECIFICATION**
